# Neurodegeneration severity can be predicted from early microglia alterations monitored *in vivo* in a mouse model of chronic glaucoma

**DOI:** 10.1242/dmm.018788

**Published:** 2015-05-01

**Authors:** Alejandra Bosco, Cesar O. Romero, Kevin T. Breen, Alexis A. Chagovetz, Michael R. Steele, Balamurali K. Ambati, Monica L. Vetter

**Affiliations:** ^1^ Department of Neurobiology and Anatomy, University of Utah, Salt Lake City, UT 84132, USA; ^2^ Department of Human Genetics, University of Utah, Salt Lake City, UT 84132, USA; ^3^ Department of Ophthalmology and Visual Sciences, University of Utah, Salt Lake City, UT 84132, USA

**Keywords:** Microglia activation, Microgliosis, Retinal ganglion cells, Confocal ophthalmoscopy, Live image analysis, *Cx3cr1*^GFP^*^/+^* DBA/2J

## Abstract

Microglia serve key homeostatic roles, and respond to neuronal perturbation and decline with a high spatiotemporal resolution. The course of all chronic CNS pathologies is thus paralleled by local microgliosis and microglia activation, which begin at early stages of the disease. However, the possibility of using live monitoring of microglia during early disease progression to predict the severity of neurodegeneration has not been explored. Because the retina allows live tracking of fluorescent microglia in their intact niche, here we investigated their early changes in relation to later optic nerve neurodegeneration. To achieve this, we used the DBA/2J mouse model of inherited glaucoma, which develops progressive retinal ganglion cell degeneration of variable severity during aging, and represents a useful model to study pathogenic mechanisms of retinal ganglion cell decline that are similar to those in human glaucoma. We imaged CX3CR1^+/GFP^ microglial cells *in vivo* at ages ranging from 1 to 5 months by confocal scanning laser ophthalmoscopy (cSLO) and quantified cell density and morphological activation. We detected early microgliosis at the optic nerve head (ONH), where axonopathy first manifests, and could track attenuation of this microgliosis induced by minocycline. We also observed heterogeneous and dynamic patterns of early microglia activation in the retina. When the same animals were aged and analyzed for the severity of optic nerve pathology at 10 months of age, we found a strong correlation with the levels of ONH microgliosis at 3 to 4 months. Our findings indicate that live imaging and monitoring the time course and levels of early retinal microgliosis and microglia activation in glaucoma could serve as indicators of future neurodegeneration severity.

## Abstract (precis)

**Highlighted Article:** This study provides the first evidence that *in vivo* monitoring of the time course and dynamics of early microglia alterations might serve as sensitive predictors of late chronic neurodegeneration in a mouse model of inherited glaucoma.

## INTRODUCTION

The ability to detect and monitor a neurodegenerative disease soon after its onset and to anticipate its future progression is a fundamental step towards uncovering early pathogenic mechanisms and developing targeted therapies. Such an early diagnostic and prognostic strategy depends on the detection of cellular and/or molecular markers dynamically linked with the pathogenic process of neurodegeneration. Growing evidence indicates that a wide range of neurodegenerative diseases are associated with innate immune responses from microglia, and in certain contexts, from recruitment of blood-derived monocytes or macrophages (Amor et al., 2014, 2010; Block et al., 2007; Cunningham, 2013; Perry and Teeling, 2013).

Microglia are long-lived myeloid cells that stably inhabit the adult CNS within parenchymal and perivascular niches (Kettenmann et al., 2011; Lawson et al., 1990, 1992; Prinz et al., 2011). Functionally, they constantly interact with surrounding neurons, blood-brain barrier cells and other glia (Davalos et al., 2005; Nimmerjahn et al., 2005; Ransohoff and Cardona, 2010; Ransohoff and Perry, 2009; Tremblay et al., 2010; Wake et al., 2009). Stress or damage to surrounding cells causes rapid microglial activation (Kettenmann et al., 2011; Kreutzberg, 1996), as identified by complex molecular, functional and cellular changes, as well as microgliosis, which refers to the expansion of microglial cell numbers by local self-renewal and/or recruitment of monocytes and/or macrophages from the blood-stream or, potentially, from latent progenitors (Ajami et al., 2007; Elmore et al., 2014; Lawson et al., 1992; Ransohoff and Cardona, 2010; Solomon et al., 2006; Streit et al., 1999). Thus, microglia, as ubiquitous, dynamic sensors of CNS damage and dyshomeostasis, are ideally suited to detect and indicate the progression of pathogenic processes.

Microgliosis and microglial activation mirror the course of neurodegeneration in both clinical and animal model studies of multiple diseases, such as Alzheimer's, Parkinson's and Huntington's disease (Ajami et al., 2011; Maeda et al., 2011; Ouchi et al., 2005; Sapp et al., 2001). Moreover, live imaging studies that have monitored microglial alterations have found evidence for their involvement at preclinical disease stages (Ajami et al., 2011; Davalos et al., 2012; Fuhrmann et al., 2010; Maeda et al., 2011; Ouchi et al., 2005; Sapp et al., 2001). Thus, CNS-resident microglia and infiltrating monocytes and macrophages are emerging as promising sensitive indicators of neuronal decline; however, their ability to predict later disease is not well defined, particularly at early stages of disease. Detection of microglial distribution and activation by molecular imaging of the brain using positron emission tomography has underscored the relevance of microglial activation as a relevant biomarker of disease in multiple neurological and psychiatric conditions (Jacobs and Tavitian, 2012; Politis et al., 2012; Venneti et al., 2013). The actual behavior of microglia at the cellular level during health, acute injury and chronic neurodegeneration has been studied in animal models over the course of minutes, and for up to 4 months, by direct observation of fluorescently labeled cells using two-photon confocal imaging of the brain, retina and spinal cord (Davalos et al., 2005, 2012; Dibaj et al., 2011; Evans et al., 2014; Fuhrmann et al., 2010; Kozlowski and Weimer, 2012; Nayak et al., 2012; Nimmerjahn et al., 2005; Tremblay et al., 2010; Wake et al., 2009), and by confocal scanning laser ophthalmoscopy (cSLO) of the retina (Maeda et al., 2014; Seeliger et al., 2005). Given that *in vivo* imaging of the retina using cSLO is non-invasive, this technique has been used to directly visualize GFP-labeled retinal microglia in the mouse eye, and in some cases to monitor changes in a single animal over a limited period of time (Alt et al., 2012; Eter et al., 2008; Liu et al., 2012; Paques et al., 2010, 2006). Although these previous studies have detected changes in microgliosis in response to acute injury, what remains to be determined is whether this approach can be used in a chronic, progressive, age-related model of retinal disease to detect and quantify early changes in microglia that would predict the later course of neurodegeneration.

**TRANSLATIONAL IMPACT**

**Clinical issue**

Specific neuronal populations progressively deteriorate in neurodegenerative diseases. Early detection is key for slowing or halting neuronal decline and loss, underscoring the importance of defining reliable indicators of disease onset and progression. The course of neurodegeneration is mirrored by innate immune responses of CNS-resident microglia and infiltrating monocytes. Live imaging studies have detected early microglial alterations in animal models and humans, suggesting that they have potential value as sensitive indicators of disease progression. However, whether the early changes of innate immune cells are related to and can predict later neurodegeneration is unknown. Glaucoma results in progressive neurodegeneration of retinal ganglion cells (RGCs) and their axons in the optic nerve and retina. The disease is mostly asymptomatic at early stages and is detectable only once vision is irreversibly compromised. Despite this, the possibility of live monitoring retinal microglia to predict future neurodegeneration severity in glaucoma has not yet been explored. Confocal ophthalmoscopy (cSLO) allows for direct and repeated visualization and tracking of the microglial alterations that precede neurodegeneration in the intact retinal niche.

**Results**

Here, the authors use a mouse model of inherited glaucoma that, as in the human condition, develops age-dependent and variably severe neurodegeneration, coupled with innate immune responses. To assess the kinetics of early microgliosis (accumulation of microglia at the site of injury) and microglia activation, they used a DBA/2J reporter substrain in which microglia and infiltrating monocytes express green fluorescent protein (GFP) under the control of the fractalkine receptor locus (*Cx3cr1*), and imaged young mice at 1 to 5 months of age by cSLO to monitor early microglial changes. The authors then aged the mice to 10 months and analyzed optic nerve pathology *ex vivo*. Across individual eyes, live images collected at pre-pathological ages revealed dynamic patterns of retinal microglial activation and an unexpected diversity in the levels of microgliosis at the optic nerve head (ONH), which was attenuated by minocycline. Notably, they identified a significant correlation between early microgliosis at the ONH and the late severity of nerve pathology.

**Implications and future directions**

This study establishes microglia as indicators and early predictors of late optic nerve degeneration in a model of chronic glaucoma. This live imaging approach is the first to visualize, quantify and monitor microglial cellular changes during early stages of a neurodegenerative disease. The findings are broadly relevant to the field of neurodegeneration because they link microglial changes to future disease progression. Moreover, this study developed a non-invasive strategy to monitor visual biomarkers that is applicable to tracking and managing glaucoma and other chronic CNS diseases that affect RGCs.

A relevant neurodegenerative disease of the retina with unresolved pathogenesis is glaucoma. The ability to detect and monitor glaucoma at early stages would offer the potential for early intervention in this disease. As a model of inherited, age-dependent glaucoma, we use DBA/2J mice, which like human patients undergo slow, asynchronous and chronic decline and loss of retinal ganglion cells (RGCs) and their axons in the optic nerve and retina (Casson et al., 2012; Nickells et al., 2012; Quigley, 1999, 2011; Weinreb and Khaw, 2004). Axonopathy first manifests at the optic nerve head (ONH), where unmyelinated axons exit the eye, and progressively expands across asymmetrical areas of the retina (Buckingham et al., 2008; Howell et al., 2007a; Inman et al., 2006; Schlamp et al., 2006; Soto et al., 2008), offering remarkable spatial resolution to track disease progression. Microglia activation has been associated with human glaucoma (Gramlich et al., 2013; Neufeld, 1999), and early alterations in retinal microglia, and potentially monocyte infiltration, precede detectable neuronal pathology in DBA/2J mice (Bosco et al., 2012, 2011; Fan et al., 2010; Howell et al., 2011). However, a predictive link between early microglia and/or peripheral monocyte alterations and later disease progression has not been defined.

To address this, our current approach took advantage of the remarkable variability of neurodegeneration severity across DBA/2J eyes (Anderson et al., 2001; Chang et al., 1999). To track retinal and ONH microglia and recruited monocytes *in vivo* with single-cell resolution, we generated DBA/2J mice carrying the microglia and monocyte reporter CX3CR1-GFP and applied recently optimized cSLO imaging and automated morphometric cell analysis to quantitatively monitor microgliosis and microglia activation from 1 to 5 months of age in a large cohort of young mice (Bosco et al., 2015). After allowing these same mice to age, we analyzed optic nerve pathology *ex vivo*. We provide evidence that microglial alterations are selectively detectable in eyes that progress to glaucoma at later stages, and establish that early microgliosis has a strong correlation with the severity of optic nerve degeneration. Taken together, these findings provide the first evidence that *in vivo* monitoring of the time course and dynamics of early microglia might serve as sensitive predictors of late chronic neurodegeneration.

## RESULTS

### Microglia can be visualized and tracked *in vivo* throughout the retina and ONH preceding neurodegeneration in *Cx3cr1*^GFP/+^ DBA/2J mice

**Fig. 1.**
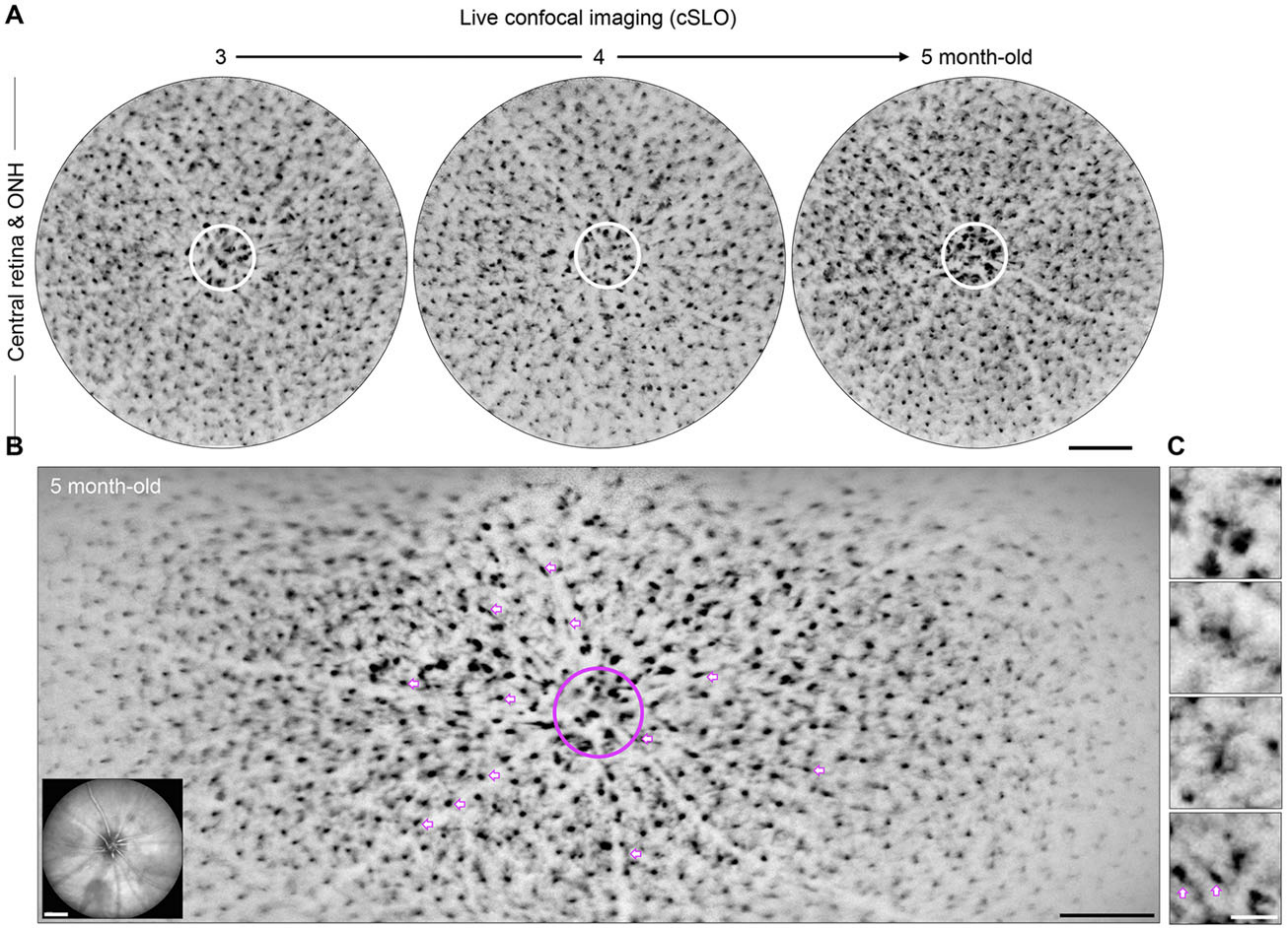
***In vivo* monthly imaging of retinal and ONH microglia and/or peripheral monocytes during early stages of chronic glaucoma.** (A) Monthly cSLO image sequence of the same eye, showing GFP^+^ cells within ∼2 mm^2^ of retina around the ONH (circle) in young *Cx3cr1^GFP/+^* DBA/2J mice. The original greyscale was inverted to improve observation. (B) Multipoint cSLO image spanning approximately one third of the same retina when the mouse was 5 months old. There is a cluster of GFP-labeled cells localized to the ONH (circle), and radial rows of perivascular cells with bipolar shape along vessels (arrows), which interrupt the regular mosaic of parenchymal microglia. Live infrared fundus images of vasculature and optic disc (inset) were acquired for each fluorescence image, to facilitate the alignment of sequential images. (C) High-magnification view of parenchymal and perivascular GFP^+^ cells (arrows). For parenchymal cells, the microglial soma size is readily identifiable, whereas process number and length are better resolved in larger cells. Scale bars: 250 µm (A,B), 50 µm (C).

To allow the long-term monitoring and quantification of the kinetics of microgliosis and microglia activation during chronic glaucoma in live DBA/2J mice, we performed backcrossing to generate a substrain carrying a knock-in allele that expresses GFP under the control of the fractalkine receptor promoter (*Cx3cr1*; Jung et al., 2000). This reporter not only labels resident microglia but also peripheral monocytes and macrophages that can infiltrate the diseased or injured CNS (Ajami et al., 2011; Broux et al., 2012; Jung et al., 2000). Using cSLO in *Cx3cr1^GFP/+^* DBA/2J mice aged 1 to 5 months, we imaged individual eyes to track GFP^+^ cells localized to the ONH and central retina (Bosco et al., 2015). At monthly intervals, we collected single and multiple *xy-*point confocal images centered on the optic disc in fundus images, and spanning approximately the central 1.5 mm or 1.5×3 to 4 mm of each retina, respectively, and its inner 30 to 40 µm in depth (Fig. 1A,B; supplementary material Fig. S1A). These live confocal images provided enough cellular resolution to visualize cell somata, and in some cases arbor dimensions (Fig. 1C), and to recognize three distinct subsets of GFP^+^ cells of microglial or macrophage origin: (1) a large population of parenchymal cells tiling the retina (200 to 300 cells), (2) a central cluster of microglia and/or blood-derived monocytes or macrophages localized to the ONH area, and (3) perivascular cells radiating from the ONH (Fig. 1B). We compared live and *ex vivo* confocal images of individual eyes collected less than 3 days apart, and confirmed that cSLO detects individual GFP^+^ cells throughout the inner retinal layers (supplementary material Fig. S1B-E). Therefore, cSLO live imaging allows the tracking of cellular changes in retinal microglia and infiltrating monocytes that are localized adjacent to RGC somata, dendrites and unmyelinated axons across the inner retinal surface and ONH (Bosco et al., 2011).

We applied cell segmentation and morphometry to cSLO images of the central 1.5-1.7 mm^2^ of retina to identify activated parenchymal microglia (Bosco et al., 2015). Briefly, using intensity-threshold-based morphometric analysis, we identified activated parenchymal microglia as cells with large somal areas (>50-60 µm^2^) and a few short processes, and non-activated parenchymal and perivascular microglia as cells with somata that were 2-3 times smaller in area and had visibly extensive and complex arbors. Microglial soma size has been shown to increase with Iba1 upregulation, and provides a metric to determine the activation status of individual GFP^+^ cells in live image analysis (Bosco et al., 2008; Kozlowski and Weimer, 2012). In sequential images of the same retinas collected at 3 and 4 months of age, we mapped and quantified numbers of activated and non-activated microglia across eight sectors radiating from the optic disc (supplementary material Fig. S2A,B), and at each age detected variable numbers of activated microglia between sectors and retinas, which were uncoupled from the concurrent changes in total GFP^+^ cell density (supplementary material Fig. S2B,C). For most of the individual retinas monitored over 2 months, numbers and distribution of GFP^+^ cells and activated microglia were dynamic (supplementary material Fig. S2C, left), although some retinas maintained relatively static levels and/or patterns of activation (supplementary material Fig. S2C, right).

### Induced changes in retinal microgliosis can be monitored over time by cSLO

**Fig. 2.**
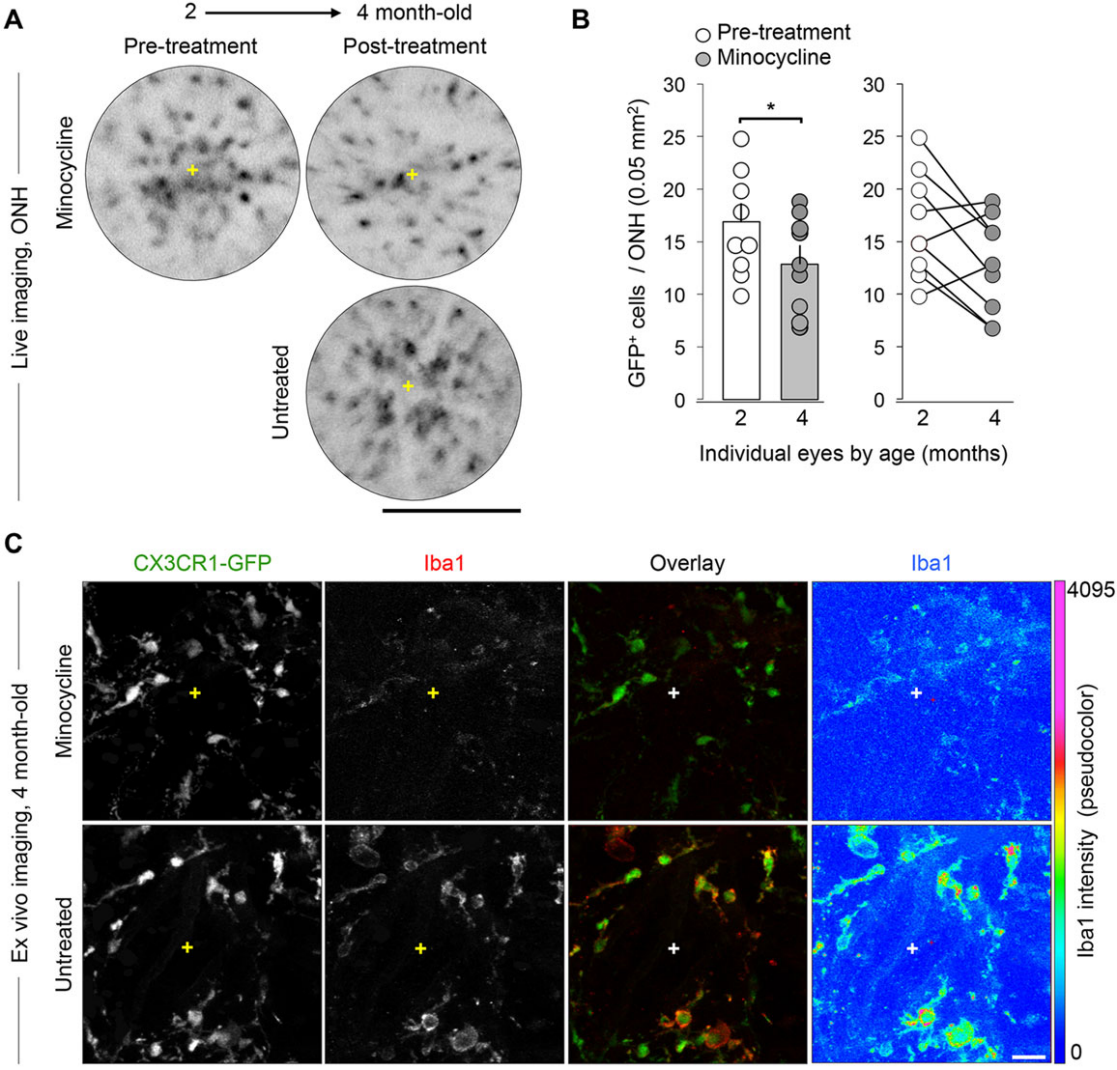
**Induced decreases in local microgliosis are detectable by live imaging.** (A) Live cSLO images of the same ONH before and after oral minocycline treatment (mouse was 2 and 4 months old, respectively) showing a reduction in GFP^+^ cell clustering, compared to an age-matched untreated control representing moderate microgliosis. (B) Total number of cells per ONH at 2 and 4 months of age (*n*=9 eyes represented by circles) plotted before and after minocycline treatment showing a significant mean reduction post-treatment (*P<0.05; Student's *t*-test). Bars indicate mean±s.e.m. at each age (16.9±1.65 cells per 0.05 mm^2^ and 13±1.53, respectively). The graph to the right depicts with lines the drop in microgliosis in 6/9 individual ONHs. (C) *Ex vivo* immunostaining of the same 4-month-old ONHs shown in A revealing that there is a noticeable downregulation of Iba1 expression, but not of GFP, after minocycline treatment, as visible in the single-channel view of Iba1 and its pseudocolor coding by expression intensity. Each image shows a maximum intensity projection of 50 µm and represents the ONH area. Scale bars: 250 µm (A), 25 µm (C).

Previous live imaging studies of retinal GFP^+^ microglia and macrophages have tracked changes in cell numbers in response to acute optic nerve injury (Eter et al., 2008; Liu et al., 2012). To determine the sensitivity of our methods to detect longitudinal changes in microgliosis in a chronic model, we induced a subtle reduction in microgliosis during early disease progression by treating young DBA/2J mice with minocycline, which we have previously shown decreases both the number and activated phenotype of retinal microglia and/or peripheral monocytes (Bosco et al., 2008). First, we identified a subset of 2-month-old mice with relatively high ONH microgliosis by cSLO imaging, then administered systemic minocycline (120 mg/kg body weight for 6 weeks), and collected cSLO images after treatment at 4 months of age (Fig. 2A). The quantification of GFP^+^ cell numbers within the ONH area showed that there were significant decreases in cell clustering in the treated eyes (*P*<0.05, *n*=9 eyes, Student's *t-*test; Fig. 2B). The ONH GFP^+^ cells also displayed a manifest reduction in Iba1 expression relative to untreated eyes (Fig. 2C), consistent with the microglial deactivation reported for minocycline in this model (Bosco et al., 2008). Thus, live retinal imaging can track an induced attenuation in early microgliosis.

### Young DBA/2J mice show highly variable levels of ONH microgliosis

**Fig. 3.**
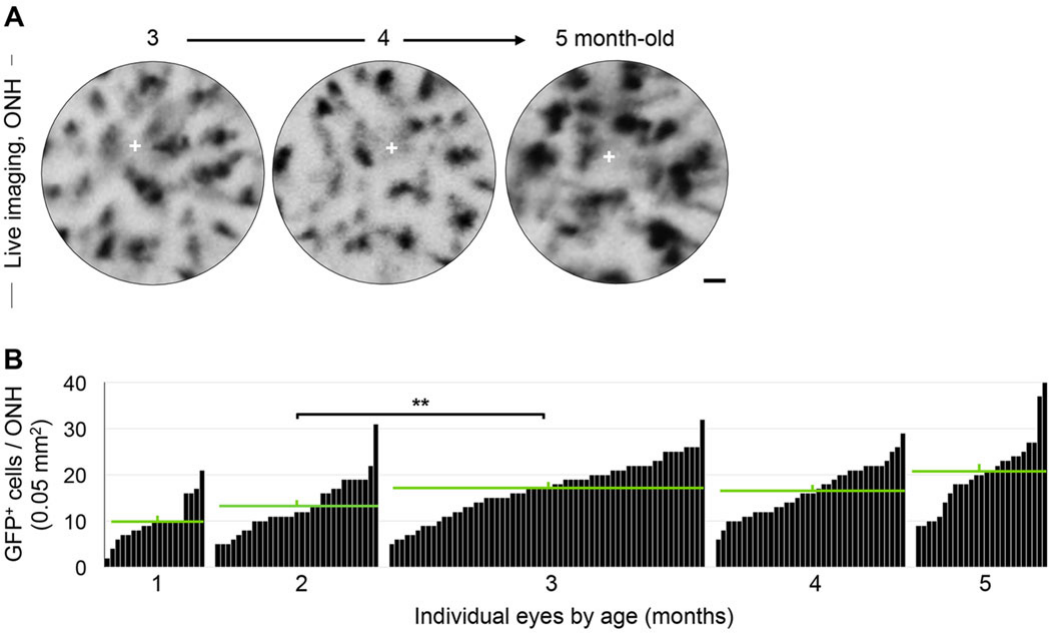
**Eyes show large variability in their levels of ONH microgliosis at pre-neurodegenerative ages.** (A) Sequential imaging of the same ONH area at 3, 4 and 5 months revealed dynamic changes in GFP^+^ cell numbers and size; the cross indicates the ONH center. (B) Total number of GFP^+^ cells per ONH at 1, 2, 3, 4 and/or 5 months of age (*n*=19-59 images per age). Data with eye identification and late nerve pathology are presented in supplementary material Fig. S3. Each bar represents an individual ONH and green bars indicate mean±s.e.m. at each age group (10±1.06, 13.17±1.07, 16.85±0.77, 16.46±0.94 to 19.93±1.53, respectively). The average number of GFP^+^ cells per ONH rises with age and significantly increases between 2 and 3 months (**P<0.01; Student's *t*-test). Scale bar: 25 µm.

Previous studies have established that DBA/2J mice typically lack histologically detectable retinal and optic nerve pathology in the first 6 months of age (John et al., 1998); however, young mice show clustering, activation and proliferation of Iba1+ microglia and/or peripheral monocytes at the ONH in some eyes (Bosco et al., 2012, 2011). Given that the age of glaucoma onset and progression are diverse in this model (Anderson et al., 2001; Chang et al., 1999), it was unclear whether these early microglia and infiltrating monocyte changes were linked to later neurodegeneration. To track the presence and levels of early ONH microgliosis prior to RGC degeneration, we analyzed cSLO retinal images collected between 1 and 5 months of age with preferential imaging at 3 months of age (Fig. 3A; supplementary material Fig. S1), when microgliosis in the DBA/2J retina peaks (Bosco et al., 2011). We detected a significant increase in the mean number of GFP^+^ cells localized to the ONH at 3 months (*P*<0.01, *n*=19 to 59 eyes; Student's *t-*test), which persisted through 5 months (Fig. 3B), in agreement with *ex vivo* studies (Bosco et al., 2011). Furthermore, statistical analysis controlling for the potential effect of repeated imaging on individual eyes confirmed this result (linear mixed model; http://CRAN.R-project.org/package=lme4; *P*<0.0001), demonstrating that the effect of repeated observations on individual eyes is negligible (*P*=0.0942). Notably, we observed that eyes of the same age displayed widely variable GFP^+^ cell density, ranging from 5 to 30 cells per 0.05 mm^2^.

### Early ONH microgliosis precedes late optic nerve degeneration

**Fig. 4.**
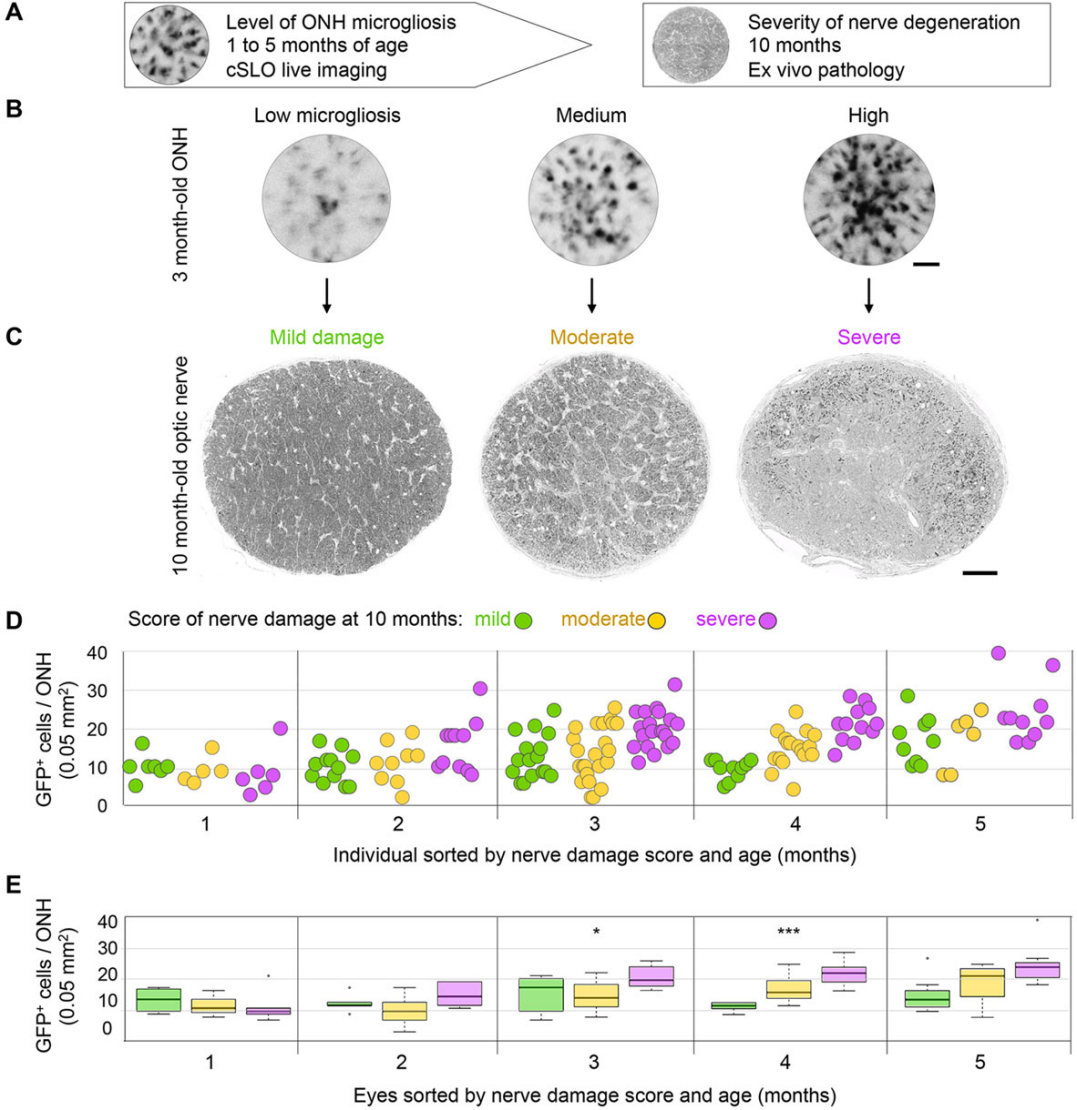
**Late nerve damage is preceded by early microgliosis at the ONH.** (A) Experimental design. (B) Live cSLO images of GFP^+^ cells localized to the ONH at 3 months of age in *Cx3cr1*^+/GFP^ DBA/2J mice showing examples of low, medium and high levels of microgliosis. (C) Light-microscopy images of optic nerve cross-sections at 10 months of age for these same eyes, representative of mild, moderate and severe damage, as assessed by visual scoring. (D) Microgliosis level, quantified as total number of GFP^+^ cells per ONH at 1 to 5 months of age (*n*=19, 31, 60, 36 and 25 eyes per respective age group), plotted for individual eyes and categorized by their corresponding optic nerve damage score at 10 months of age (color-coded as indicated). (E) Box plots of the same dataset illustrate significant changes at 3 and 4 months of age (*P<0.05 and ***P<0.001, respectively; Kruskal–Wallis rank ordered test), most notably in the severe nerves at both ages, and in the moderate nerves at 4 months. Plots indicate the median (thick line), interquartile range (IQR; box height), and data within 1.5 times the IQR (whiskers) or greater (outliers, circles). Scale bars: 250 µm (A) and 50 µm (C).

The diversity in levels of ONH microgliosis raised the question as to whether it reflects early, differential progression towards disease in a subset of eyes. To address this possibility, we assessed optic nerve pathology *ex vivo* in the same eyes at 10 months of age, when glaucomatous degeneration is evident in many DBA/2J mice (Fig. 4A-C). Glaucoma onset and the severity of neurodegeneration are highly variable in DBA/2J mice, thus axonal pathology can manifest with variable levels and distribution in individual optic nerves (Buckingham et al., 2008; Howell et al., 2007a; Inman et al., 2006; Jakobs et al., 2005; John et al., 1998; Libby et al., 2005; Schlamp et al., 2006; Soto et al., 2008). Using an established damage scoring system (Anderson et al., 2005; Howell et al., 2007a; Libby et al., 2005), high-resolution light microscopy images of 1-µm-thick cross-sections of individual nerves were categorized at one of three levels of damage based on qualitative parameters of pathology (Fig. 4C; see Materials and Methods). Next, the numbers of GFP^+^ cells per ONH at 1 through to 5 months of age were plotted for nerves in each damage category (Fig. 4D). All 1-month-old mice showed similarly low GFP^+^ cell densities in the ONH, but this analysis revealed a distinct trend to increase from 2 to 5 months of age, where nerves with severe late damage showed the highest levels of microgliosis, whereas nerves with moderate damage were preceded by more intermediate and variable levels of microgliosis. A Kruskal–Wallis rank ordered test showed that the median GFP^+^ cell density per ONH for each level of severity of optic nerve pathology was significantly different at 3 months (χ^2^=07.84, *P=*0.0199) and 4 months of age (χ^2^=16.27, *P=*0.0003), and marginally significant at 5 months of age (Fig. 4E). This initial analysis shows a significant relationship between the early number of GFP-labeled cells at the ONH and the extent of late optic nerve degeneration.

### Early ONH microgliosis correlates with future optic nerve pathology

**Fig. 5.**
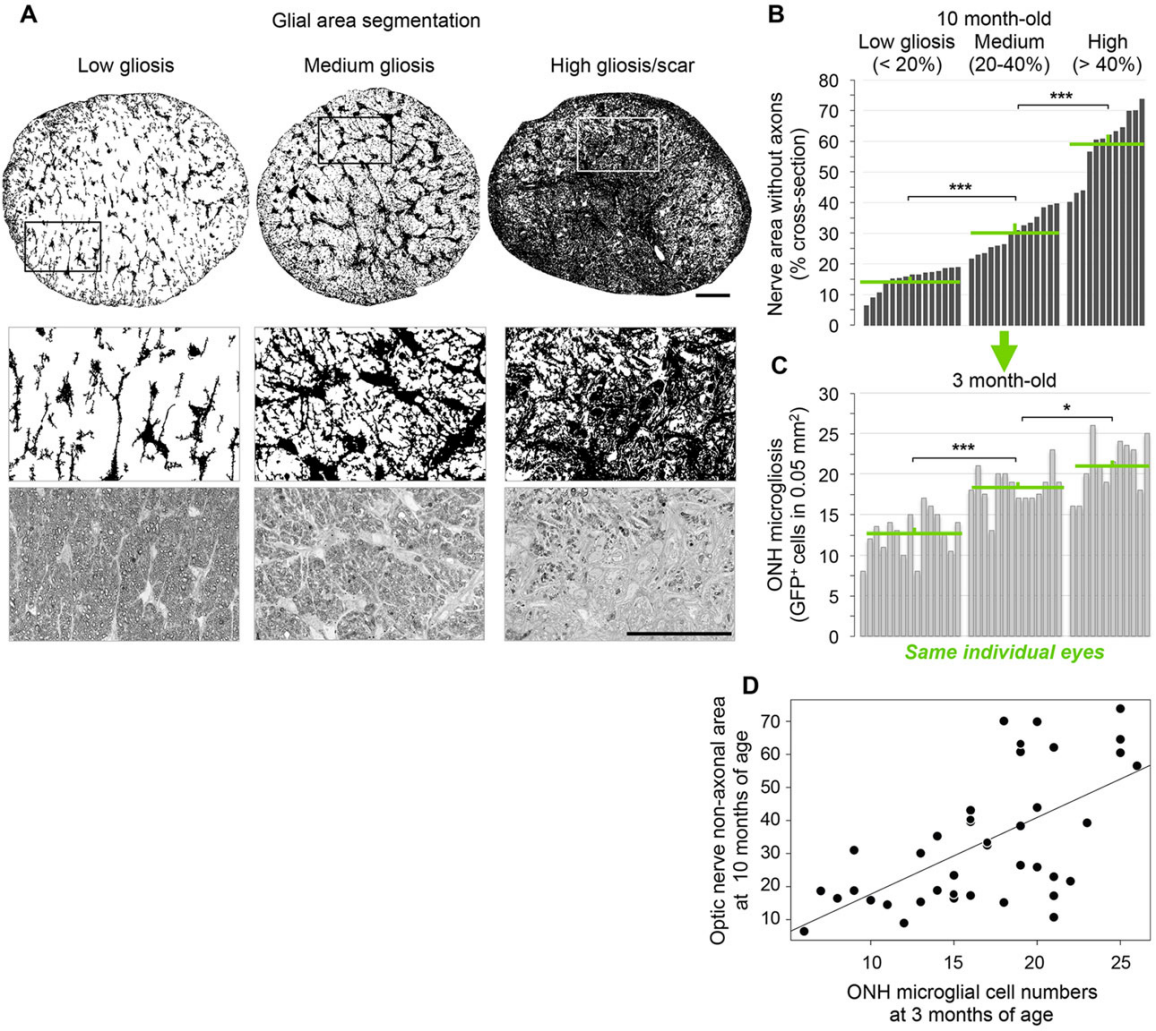
**Severity of nerve pathology and early microgliosis show a positive correlation.** (A) Threshold analysis of the nerve area occupied by glial cells or scar for the cross-sections in Fig. 4C, and high-magnification views of framed areas. (B) Plot of individual optic nerves from 10-month-old mice sorted by relative glial area and ascending coverage, and classified as low, medium and high gliosis (15.24±0.96, 30.38±1.67 and 59.05±3.22 mean±s.e.m. non-axonal area, respectively). Nerves show a spectrum of glaucomatous damage, with significant increases in mean gliosis across groups (***P<0.001; Student's *t-*test). (C) Corresponding microgliosis level or total GFP^+^ cell number per ONH at 3 months, plotted by their glial area at 10 months (i.e. same order as in B). Microgliosis is categorized by level as low, medium and high microgliosis (12.63±0.70, 18.43±0.62 and 21.08±0.97 mean cells per ONH, respectively). The mean ONH microgliosis shows significant increases across levels of nerve gliosis (***P<0.001 and *P<0.05; *n*=41 eyes; Student's *t-*test). Green lines indicate means and s.e.m. per group. (D) There is significant correlation between the optic nerve glial relative area at 10 months (non-axonal area) and their ONH microgliosis at 3 months (*P=*0.0001; Spearman's rank test). Scale bars: 50 µm.

To thoroughly define the correlation between ONH microgliosis at a young age and the susceptibility to develop glaucoma at an older age, we established a quantitative measure of optic nerve pathology based on the magnitude of gliosis and gliotic scaring associated with nerve degeneration (Crish et al., 2010; Libby et al., 2005; Schlamp et al., 2006). It is well documented that as axons are lost in the optic nerve, the area is replaced by reactive glia (hypertrophic and proliferative), and by a glial scar at the end stages of neurodegeneration (Burda and Sofroniew, 2014; Crish et al., 2010; Dai et al., 2012; Hernandez, 2000; Lye-Barthel et al., 2013; Qu and Jakobs, 2013; Sofroniew, 2009; Son et al., 2010; Sun and Jakobs, 2012; Sun et al., 2009). Therefore, the reduction of the non-axonal area at the expense of expanding glial territories in late stages of glaucoma was used as readout of nerve pathology severity. For this, we applied threshold-based segmentation of glial cells and scar tissue to high-resolution light images of entire optic nerve sections from 10-month-old mice (Fig. 5A, see Materials and Methods). Consistent with the variability in glaucoma onset and progression in DBA/2J mice, individual optic nerves showed large variability in the relative area occupied by glial cells or scar, which was consistently increased in nerves with widespread axonal damage and loss. According to the quantification of non-axonal area, individual *Cx3cr1*^GFP/+^ DBA/2J nerves were classified as healthy if their total glial territory occupied less than 20% of the nerve, which is comparable to *Gpnmb*^+/SjJ^ DBA/2J nerves (A.B., K.T.B., S.R.A., M.R.S., D.J.C. and M.L.V., unpublished data), the congenic control strain that carry a wild-type *Gpnmb* allele and do not undergo RGC degeneration (Howell et al., 2007b). Glaucomatous nerves with increased glial coverage were classified as having medium (20-40%) or high (>40%) gliosis (Fig. 5B). To confirm this classification, we compared the average glial area in each category and observed a significant increase from low to medium and then to high gliosis (*P*<0.001, *n*=41 eyes; Student's *t-*test). We thus used the expansion of gliosis and gliotic scar throughout areas devoid of RGC axons as a novel and objective measure of nerve degeneration severity.

Plotting the levels of early ONH microgliosis versus the magnitude of late nerve gliosis for each individual eye (Fig. 5C) revealed that nerves with low glial area or mild damage at 10 months showed low ONH microgliosis at 3 months. In contrast, eyes with moderate or severe nerve degeneration, evident by extensive gliosis or glial scaring, were preceded by intermediate and high levels of early ONH microgliosis, respectively. The comparison of the mean ONH microgliosis for eyes grouped by late nerve pathology revealed significant increases across the three levels of gliosis (*P*<0.001 and *P<*0.05, *n*=41 eyes; Student's *t-*test). A Spearman's rank-ordered correlation showed that nerve glial area at 10 months was significantly correlated with the number of microglial cells observed at 3 or 4 months (*rho*=0.56, *P=*0.0001; Fig. 5D). These analyses establish a predictive link between early microglia and/or peripheral monocyte alterations and later disease progression.

### Resident microglia are main components of early ONH and retinal microgliosis

**Fig. 6.**
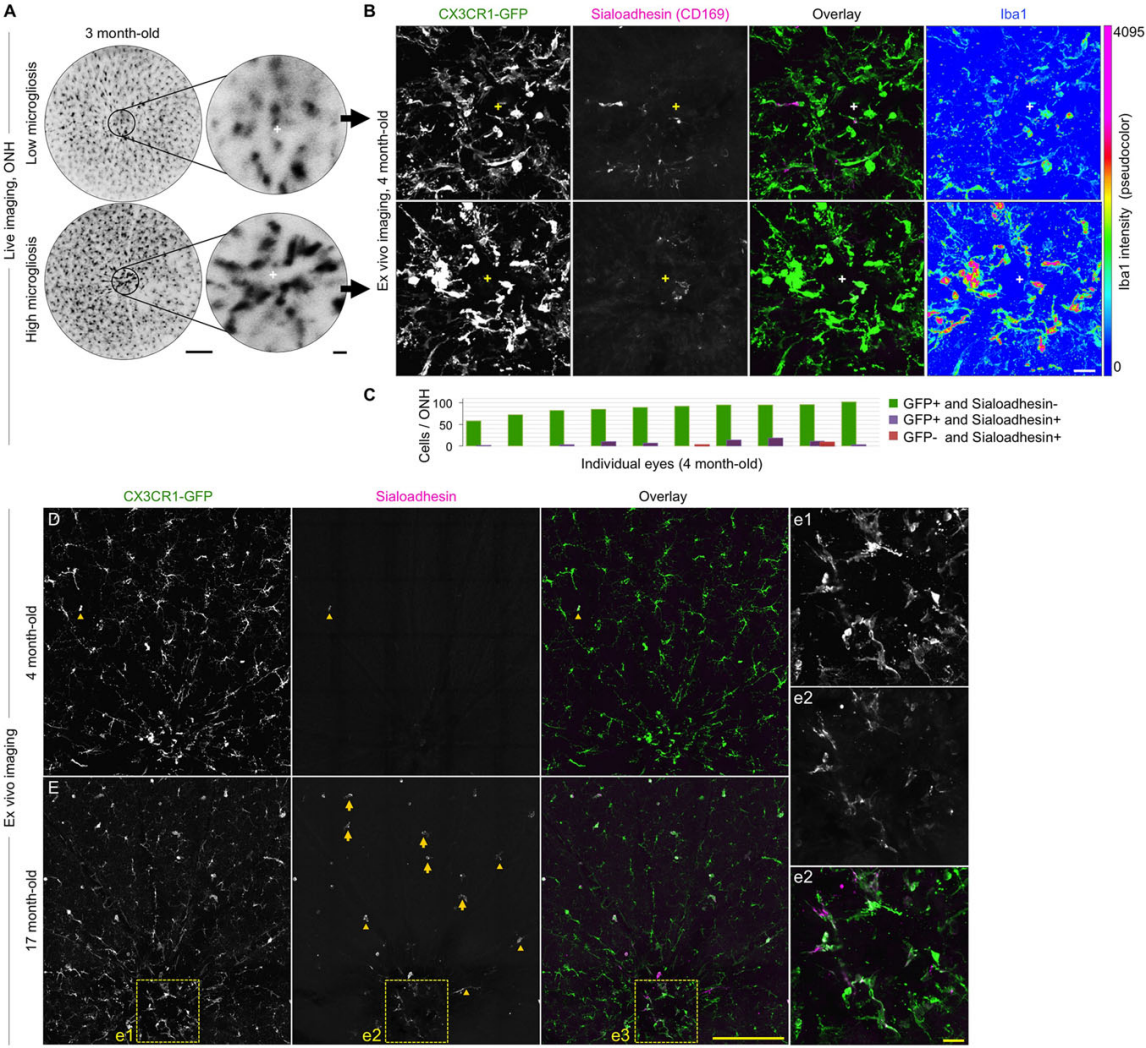
**Early microgliosis is mainly driven by microglia resident in the ONH.** (A) Live cSLO images of two 3-month-old retinas (left) and their ONHs (right, cross indicating its center), representative of low and high microgliosis. (B) *Ex vivo* confocal images of the same ONHs at 4 months of age, shown as maximal intensity projection of the inner 30 µm. Triple-immunostaining detects few cells that are positive for both GFP and sialoadhesin within the ONH, regardless of their different levels of microgliosis and activation, revealed by upregulated Iba1 expression in the ONH with increased microgliosis. (C) Number of cells expressing GFP and/or sialoadhesin per ONH area at 4 months of age. Single- and double-stained cells were quantified in confocal images spanning the central 200 µm of retinal whole mounts, throughout the inner 60 µm (0.8-µm *z*-slices). (D) Low magnification, single-slice view of the same 4-month-old retina representative of high microgliosis (B, bottom row). (E) Comparable view of a 17-month-old retina, showing sialoadhesin expression in perivascular (arrowheads), parenchymal (arrows) and ONH cells (frame). The ONH area is shown at higher magnification in corresponding insets. Scale bars: 250 µm (A,B,D and E), 25 µm (insets in A, and e).

The selective increase in microgliosis detected prior to optic nerve degeneration in DBA/2J mice could involve resident microglia and/or proinflammatory monocyte entry to the ONH, as recently suggested (Howell et al., 2012). To address whether the early microgliosis detected by cSLO can be attributed to infiltrating cells, we assessed *ex vivo* the expression of sialoadhesin (Siglec-1 or CD169), which identifies infiltrating monocytes and macrophages, as well as vitreal hyalocytes and dendritic cells, but is absent in resident microglia (Butovsky et al., 2012; Davies et al., 2013; Vagaja et al., 2012). Sialoadhesin-expressing cells are absent in the brain and retina under physiological conditions, where the blood-brain or blood-retina barrier is intact (Hartnell et al., 2001; Linnartz-Gerlach et al., 2014; Perry et al., 1992; Sancho-Pelluz et al., 2008). Immunostaining of retinal whole mounts at 4 months of age detected only scarce sialoadhesin-expressing cells, and their numbers were independent of the relative levels of ONH microgliosis detected by live imaging at 3 months (Fig. 6A,B). Furthermore, quantification of cells expressing sialoadhesin in the ONH area at 4 months of age (*n=*10 retinas; Fig. 6C) detected no sialoadhesin in 86.60% of CX3CR1-GFP^+^ cells, colocalization of GFP and sialoadhesin in 7.39% of cells, and 0.58% of cells expressing sialoadhesin but not GFP (in only two out of ten retinas). Consistent with previous reports of monocyte infiltration (Howell et al., 2012), we detected an increased presence of cells that were positive for both sialoadhesin and GFP throughout the central retina in 17-month-old DBA/2J mice (*n*=4); these cells were localized to the ONH, the retinal parenchyma and along blood vessels; however, such cells were infrequent at 4 months of age (Fig. 6D,E). Overall, these results reveal the presence of a small subset of peripheral monocytes confined to the ONH area in young DBA/2J mice, and point to resident microglia as the main cells underlying microgliosis during early stages of glaucoma progression.

## DISCUSSION

Activation of microglia and innate immunity responses have been linked to the early stages of neurodegeneration (Ajami et al., 2011; Amor et al., 2014, 2010; Block et al., 2007; Cunningham, 2013; Davalos et al., 2012; Fuhrmann et al., 2010; Maeda et al., 2011; Ouchi et al., 2005; Perry and Teeling, 2013; Sapp et al., 2001). In the retina, microglia activation is also associated with early stages of disease in animal models of chronic glaucoma (Bosco et al., 2012, 2011; Howell et al., 2011), as well as age-related macular degeneration (Combadière et al., 2007; Gupta et al., 2003; Karlstetter and Langmann, 2014; Sennlaub et al., 2013). However, it has been unclear whether these early responses are indicative of later severity or patterns of neurodegeneration. Here, we addressed this using the DBA/2J model of glaucoma, a chronic model of retinal neurodegeneration that is variable in onset and progression (Anderson et al., 2001; Chang et al., 1999). Building upon previous studies of direct *in vivo* imaging of CX3CR1-GFP^+^ retinal microglia and infiltrating monocytes using cSLO in models of acute injury (Alt et al., 2012; Eter et al., 2008; Liu et al., 2012; Paques et al., 2010, 2006), and morphometric quantification of microglia activation in live confocal images (Bosco et al., 2015; Kozlowski and Weimer, 2012), we performed live imaging at early disease stages, then allowed the animals to age and assessed late RGC pathology in the optic nerve.

We found that early microgliosis within the ONH is strongly correlated with later severity of optic nerve degeneration. The detection of early microgliosis is consistent with previous *ex vivo* studies that have reported microglia and/or peripheral monocyte clustering, activation and proliferation at the ONH prior to detectable neuronal pathology in this model (Bosco et al., 2011), as well as early infiltration of proinflammatory monocytes (Howell et al., 2012). Gene expression studies have also identified the activation of pathways related to innate immune responses and monocyte recruitment during early progression of chronic glaucoma (Fan et al., 2010; Howell et al., 2012; Steele et al., 2006). Previous studies tracking GFP^+^ microglia by cSLO after induction of acute damage to RGCs, by optic nerve crush or intraocular pressure elevation, have detected increases in retinal microgliosis that were associated with reduced survival of RGCs (Alt et al., 2012; Liu et al., 2012). Here, using a chronic model of glaucoma, we found early microgliosis clustered at the ONH, and were able to link this to the later severity of optic nerve degeneration. Our findings suggest that ONH microgliosis might be an early indicator of RGC stress or damage, consistent with the localization of the earliest axonal damage to the retina-nerve interface (Howell et al., 2007a; Soto et al., 2008).

### Microglia resident in the unmyelinated optic nerve and central retina initiate focal microgliosis

Proinflammatory, circulating monocytes infiltrate the DBA/2J ONH and have been observed in 10.5-month-old animals (Howell et al., 2012). At young ages, our preliminary analysis suggests that microglia represent the majority of the CX3CR1-GFP^+^ cell population contributing to early microgliosis within the ONH and central retina, because there are few cells that express sialoadhesin (Siglec-1 or CD169), a marker of peripheral monocytes and/or macrophages, as well as of dendritic cells (Asano et al., 2011; Butovsky et al., 2012; Hartnell et al., 2001; O'Neill et al., 2013), although it is possible that sialoadhesin expression only identifies a subset of retinal and ONH peripheral monocytes. Interestingly, we find in 17-month-old DBA/2J retinas that sialoadhesin is expressed in the majority of amoeboid monocyte-lineage cells that are abundant within the ONH and throughout the retina, and which have been attributed to monocyte infiltration in previous studies (Howell et al., 2012). Thus, we suggest that early microgliosis is mainly driven by changes in resident microglia, both in the ONH and retina. Given the importance of proinflammatory monocyte infiltration in neurodegeneration (Conductier et al., 2010; Howell et al., 2012; Naert and Rivest, 2013; Perry and Teeling, 2013; Ransohoff and Cardona, 2010; Schwartz et al., 2013), it will be important to explore the crosstalk between microglia and infiltrating monocytes and/or macrophages during early versus late disease progression in glaucoma.

The pigmentary form of glaucoma in DBA/2J mice depends upon mutations in the two melanosomal proteins TYRP1 (tyrosinase-related protein 1) and GPNMB (glycoprotein nmb) (Anderson et al., 2001). We have previously shown that early clustering and activation of microglia was not evident in mice of the congenic substrain DBA/2J *Gpnmb*^+/SjJ^ (Howell et al., 2007b). Notably, GPNMB is also expressed in cells of the macrophage and microglia lineage, and functions as a repressor of inflammation (Huang et al., 2012; Libby et al., 2005; Mo et al., 2003; Ripoll et al., 2007; Zhou et al., 2005). Thus, the early microglial changes documented in the DBA/2J mouse (Bosco et al., 2015, 2012, 2011; Fan et al., 2010) could reflect intrinsic immune components, or immune responses to early stress or damage in RGCs that might contribute to glaucoma pathogenesis. Nevertheless, ONH and central retinal microglial alterations represent the earliest retinal change evident in the DBA/2J mouse, and are strongly correlated with later disease. Although intraocular pressure is not consistently elevated at young ages in DBA/2J mice, progressive functional decreases have been detected after 3 months of age (Saleh et al., 2007). Thus early changes in RGC function are apparent, consistent with the early changes that we see in microglia at these ages.

### Live, long-term visualization of microglial changes during neurodegeneration progression and treatment

We conclude that monitoring microglia activation and microgliosis at prepathological stages might be a powerful tool for detecting the onset and tracking progression of neurodegenerative disease. The retina is an ideal region of the CNS to achieve this because specific cell populations can be directly and repeatedly visualized in the intact organism by the use of cSLO and cell-selective transgenic labels (Alt et al., 2012; Chauhan et al., 2012; Eter, 2010; Eter et al., 2008; Kumar and Zhuo, 2010; Leung et al., 2008a,b; Leung and Weinreb, 2009; Liu et al., 2012; Nakano et al., 2011; Paques et al., 2010, 2006; Schallek et al., 2013; Schön et al., 2012; Seeliger et al., 2005). Previous live imaging studies have used the *Cx3cr1*^GFP/+^ transgenic label for cSLO imaging in acute models of induced RGC injury (Alt et al., 2012; Liu et al., 2012). Here, we applied this to a chronic progressive neurodegenerative disease, and provided quantitative analysis of cell changes in the context of chronic neurodegeneration, by extending the period of tracking for microglial and/or peripheral monocytic changes over 5 months, and then analyzing disease progression at 10 months. The tracking of early microglial changes in young DBA/2J mice offers the possibility of optimizing the use of this chronic model by allowing the selection of young animals in which one or both eyes will likely progress to severe glaucoma at advanced ages, thus reducing the inter-individual variability in disease progression within experimental samples. This strategy should advance the already intense studies aimed at elucidating the complex cellular and molecular mechanisms that underlie neurodegeneration in chronic glaucoma (Anderson et al., 2006; Howell et al., 2011; Jakobs, 2014; Nair et al., 2014; Rieck, 2013; Steele et al., 2006), and identify initiating events associated with disease onset or early progression potentially relevant to glaucoma patients.

Other imaging modalities allow quantitative detection of regional microglia activation *in vivo* in chronic CNS pathologies, including positron emission tomography of radioligands for microglia and astrocyte translocator protein (TSPO) receptors, as well as magnetic resonance and bioluminescence imaging of neuroinflammation (Jacobs and Tavitian, 2012; Trapani et al., 2013; Venneti et al., 2013). However, *in vivo* visualization of microglia by cSLO provides cellular resolution, which enables quantitative analysis of microglia activation based on somal size (Bosco et al., 2015), similar to analysis of brain microglial activation in two-photon confocal images (Kozlowski and Weimer, 2012). We observed highly dynamic and sectorial patterns of microglia activation within the retinal parenchyma, suggesting that microglia might be responsive to local changes within the retina during early disease stages. Given that these changes are complex, this possibility will be addressed in future studies using early markers of neuronal stress or dysfunction. Our assessment of microglial behavior near glaucoma onset demonstrates the power of *in vivo* retinal imaging to detect early progression of neurodegeneration at the cell level, with high spatial and temporal resolution.

We also establish the feasibility of visually tracking induced changes in retinal microglia activation to assess therapies, in this case by administering minocycline to decrease activation. With the development of non-genetic labels for microglia, this could be a potential tactic applicable to both glaucoma management and treatment evaluation. Future studies could also evaluate the possibility of detecting retinal microglia and/or peripheral monocyte changes resulting from diverse pathologies because the retina and optic nerve are targeted for neurodegeneration in diseases such as Alzheimer's, Parkinson's and multiple sclerosis (Chan, 2012; Frost et al., 2010; Guo et al., 2010; Ikram et al., 2012; Kersten et al., 2014; Kesler et al., 2011). In line with this, the use of live detection of retina and ONH pathology for early Alzheimer's disease management is under intense study (Ikram et al., 2012; Petzold et al., 2010; Satue et al., 2014). Overall, our findings suggest that retinal microglia might serve as sensitive neuroimaging biomarkers to detect early and/or unidentified stages of disease onset and progression in glaucoma, and potentially in other neurodegenerative diseases that impact the retina and optic nerve.

## MATERIALS AND METHODS

### Mice

Heterozygote *Cx3cr1*^GFP/+^ DBA/2J mice were derived by backcrossing homozygous C57BL/6.129P-*Cx3cr1*^tm1Litt^/J mice, which express GFP under the control of the fractalkine receptor locus, for more than ten generations (Jung et al., 2000) with DBA/2J mice (Jackson Laboratories, Bar Harbor, ME). All mice were bred in-house, introducing new breeders every 3 to 4 generations. This study used females only. Mice were maintained and imaged in pathogen-free facilities, using protocols approved by the Institutional Animal Care and Use Committee at the University of Utah.

### cSLO *in vivo* imaging of GFP^+^ cells in the retina and ONH

Retinal and ONH GFP^+^ microglial and/or peripheral monocyte cells were monitored using confocal scanning laser ophthalmoscope (cSLO) images collected at monthly intervals in mice aged 1 to 5 months (±1 week), following a recently reported protocol (Bosco et al., 2015). Eyes with corneal or iris defects detectable by fundus imaging were excluded from this study, and imaging was discontinued after 5 months of age, when corneal and/or iris defects prevented effective pupil dilation and reliable imaging. Briefly, mice anesthetized by intraperitoneal injection of Avertin (1.3% 2,2,2-tribromoethanol and 0.8% tert-amyl alcohol, Sigma-Aldrich, St Louis, MO) and fitted with PMMA contact lenses (Cantor & Nissel Ltd., Northants, UK), were imaged unrestrained with a cSLO system equipped with a 55° wide-field lens and real-time eye tracker (Spectralis HRA+OCT, Heidelberg Engineering). The central retina was visualized in single-point images spanning an area of ∼1.5 mm in diameter, or in composite images spanning 40 to 60% of the retina (∼1.5×3-4 mm), both across the inner planes of the retina and ONH (30-40 µm axial depth; 55-60° focus), using by 820-nm or 488-nm laser excitation (460-490 nm barrier filter set) to produce fundus and fluorescence images, respectively.

### Live image analysis of cell density and morphological activation

To analyze ONH microgliosis, we manually identified and counted total GFP^+^ cells within a ∼250-µm-diameter circle centered on the optic disc in cSLO images. In the surrounding central retina, we performed automated cell and somal segmentation followed by morphometric analysis using intensity-based thresholding in cSLO images of the central retina (∼1.5 mm^2^) (Bosco et al., 2015). Briefly, individual somal areas were automatically measured, sorted into groups of activated microglia (>50-60 µm^2^), non-activated microglia (10-50 µm^2^) and manually identified cells <10 µm^2^) and mapped to eight radial sectors (∼200 µm^2^) that subdivided the central retina and excluded the ONH.

### Minocycline treatment

A subset of mice that showed high ONH microgliosis by cSLO imaging at 2 months old (*n*=9), was administered systemic minocycline (120 mg/kg body weight, Sigma-Aldrich) on weekdays (Monday-Friday) for 6 weeks, from 2.5 to 4 months of age, by oral gavage (Bosco et al., 2011).

### Mouse tissue collection

Mouse eyes were collected as previously described (Bosco et al., 2011). Briefly, under full anesthesia induced by inhalation of 2% (vol) Isoflurane in 2 liter/min oxygen, mice were transcardially perfused with 5 ml PBS followed by 20 ml 4% paraformaldehyde (PFA; Electron Microscopy Sciences, Hatfield, PA) in 0.1 M PBS circulated with a peristaltic pump (Dynamax, Rainin, Oakland, CA). Eyes with optic nerves were isolated from the brain and post-fixed for 2 h in PFA at 4°C, and nerves were dissected and further fixed overnight in 1.2% PFA and 0.8% glutaraldehyde in PB (Electron Microscopy Sciences).

### Retinal histology

Retinas, with the dorsal pole of the eyes marked with a cauterizer, were processed as previously described (Bosco et al., 2012, 2008, 2011). Whole-mount retinas were immunostained using mouse anti human phospho-neurofilament (pNF, Dako, Carpinteria, CA), rabbit anti-human Iba1 (Wako, Richmond, VA), goat anti-GFP (Abcam, Cambridge, MA), and rat anti-mouse CD169 (sialoadhesin) conjugated to Alexa Fluor 647 (MOMA-1 clone, AbD Serotec, Bio-Rad, Raleigh, NC) primary antibodies, which were detected with Alexa-Fluor-conjugated donkey anti-IgG secondary antibodies (Invitrogen, La Jolla, CA).

### *Ex vivo* confocal imaging

Confocal images spanning entire retinal flat mounts were generated as previously described (Bosco et al., 2012, 2011) using a confocal imaging system equipped with a 20× lens and a resonant scanner (A1R confocal, Eclipse Ti inverted microscope and NIS-Elements C, Nikon). Multipoint images (625 *xy* positions) were acquired at high resolution (0.41 µm/px), then stitched and projected as maximal intensity of the inner 30-40 µm of retina (0.8-µm step). To allow image analysis and quantification, the parameters of image acquisition were maintained constant between retinal samples, and, for illustration, images had their brightness and contrast minimally edited. Counts of ONH cells expressing GFP and/or sialoadhesin where manually performed in the central 200×200 µm around the optic disc, visualizing each channel independently in maximum-intensity projections, and verifying colocalization in slice view, through the *z* plane.

### Optic nerve histopathology

Nerves from 10-month-old *Cx3cr1*^GFP/+^ DBA/2J mice including the retro-orbital, myelinated segment (1-1.5 mm post-lamina), were prepared as 1-µm-thick plastic cross-sections, and stained with a modified paraphenylenediamine (PPD) protocol and Toluidine Blue (Anderson et al., 2005; Calkins et al., 2005; Inman et al., 2006; Sappington et al., 2003). High-resolution multipoint (36 *xy*) images were generated using a compound light-microscope and a 60× lens (BX51 and cellSens software, Olympus, Center Valley, PA).

### Quantification of nerve gliosis

By direct visual inspection of high-resolution multipoint images obtained from entire optic nerve cross-sections, we identified glial cells and the gliotic scar as areas lacking dystrophic or intact axons, as well as meninges and vascular lumen. To segment glial areas and vascular spaces, we applied automatic thresholds to the red RGB channel to generate a binary overlay representing axon-free nerve areas (FluoRender, University of Utah). To quantify the relative area occupied by glial cells and/or the gliotic scar, we measured the cross-sectional area of the nerve and subtracted the area corresponding to blood vessels and meninges. Random samples independently segmented and measured by two investigators showed <5% variation in measured glial area (*n*=10).

### Statistics

Statistical significance was calculated by a Student's post-hoc *t-*test (unpaired, two tailed; Fig. 2B, Fig. 3B and Fig. 5B,C), by a linear mixed model (http://CRAN.R-project.org/package=lme4; Fig. 3B) or Kruskal–Wallis rank-ordered test (Fig. 4E), and is indicated in the graphs as: *P<0.05, **P<0.01 and ***P<0.001. Correlation analysis was performed by Spearman's rank-ordered test (Fig. 5D). Analyses were conducted in R: A language and environment for statistical computing 2014 (R Core Team, R Foundation for Statistical Computing, Vienna, Austria) or Excel v. 14.4.6 (Microsoft, Redmond, WA).

## Supplementary Material

Supplementary Material
