# Supplementary material for: Neurodegeneration severity can be predicted from early microglia alterations monitored in vivo in a mouse model of chronic glaucoma
Source: Dis Model Mech. 2015 May 1;8(5):443–55. doi: 10.1242/dmm.018788 (PMC4415894; doi:10.1242/dmm.018788)
Supplement: Supplementary Material [file supp_8_5_443__index.html]

Neurodegeneration severity can be predicted from early microglia alterations monitored in vivo in a mouse model of chronic glaucoma — Supplementary Material 

# Neurodegeneration severity can be predicted from early microglia alterations monitored *in vivo* in a mouse model of chronic glaucoma

## DMM018788 Supplementary Material

**Files in this Data Supplement:**

- **Supplementary Material**
