# Supplementary material for: Neurodegeneration severity can be predicted from early microglia alterations monitored in vivo in a mouse model of chronic glaucoma
Source: Dis Model Mech. 2015 May 1;8(5):443–55. doi: 10.1242/dmm.018788 (PMC4415894; doi:10.1242/dmm.018788)
Supplement: Supplementary Material [file supp_8.5.443_DMM018788.pdf]

SUPPLEMENTAL FIGURE LEGENDS

Suppl. Fig 1. cSLO renders detailed images of GFP+ cells localized to a large retinal area in CX3CR1$^{GFP/+}$ DBA/2J mice. (A) Sequence of representative composite cSLO images (same retinas as Fig.1A), with corresponding fundus images. (B) Infrared image of the central retina, showing the vitreal vasculature and optic disc/ONH area (circle) in a 3 month-old mouse. (C) Corresponding cSLO images of GFP+ cells, shown as the original image and with inverted greyscale. (D) Ex vivo confocal images of the same retina as in B, viewed as maximal intensity projection of 30-40 µm below the vitreal surface for the entire retina (top) and for the central retina area, comparable to the live cSLO images shown in C. Comparison of ex vivo images collected 1 to 3 days after live imaging (n = 15) allowed us to define the depth of live images, which spanned the inner 30-40 µm of retina including the nerve fiber layer, ganglion cell layer and inner plexiform layer. (E) Many individual GFP+ cells could be clearly recognized by both imaging methods, although with relatively finer detail ex vivo. Scale bars: 250 (A-C) and 2,500 µm (D).

Suppl. Fig. 2. Retinal maps of early microglia activation and microgliosis show variable and dynamic patterns. (A) Live image analysis showing the threshold, segmentation of individual GFP+ cell and somata and the taxonomy of somal areas to distinguish activated cells (magenta, somal area > 50-60 μm^2) from non-activated cells (blue, somal area < 50 μm^2), corresponding to a cSLO image acquired at 5 months of age and shown in Figure 1A. (B) Sequential cSLO images of a single retina, acquired at 3 and 4 months of age, showing the central third of the retina divided in 8 radial sectors (200 μm^2 each), (C) Sectorial analysis of numbers of total GFP+ cells and activated microglia (somal area > 50-60 μm^2) for 6 retinas, which show variable patterns of GFP+ cell changes from 3 to 4 months of age (left), or stable sectorial patterns (right). Scale bars: 250 (A and B).

Suppl. Fig. 3. Detailed dataset corresponding to Figure 3B and 4D, E). Total number of GFP$^+$ cells per individual ONH, showing ages quantified per individual eyes between 1 and 5 months of age. Individual eyes are grouped according to their optic nerve damage scored at 10 months of age. The longitudinal imaging of mice between 1 and 5 months of age (*n* = 30) produced image sequences for individual eyes, which were amenable for quantification at all 5 months (*n* = 8 eyes), or over 4, 3, 2 months of age (*n* = 9, 10, 16 eyes, respectively). The graph background was greyed for every other eye for easier identification of individual longitudinal imaging sets.

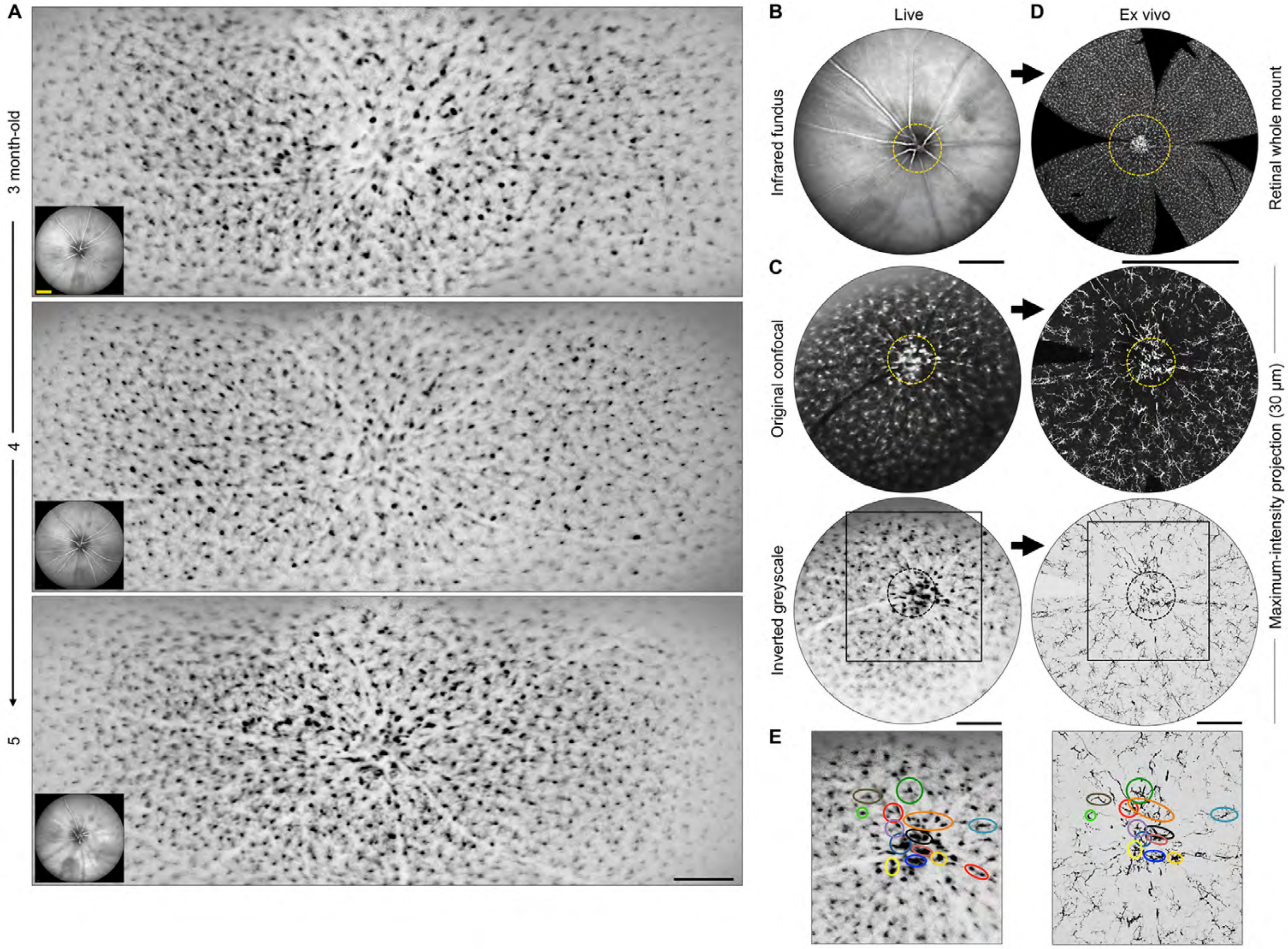

Supplementary Figure 1.

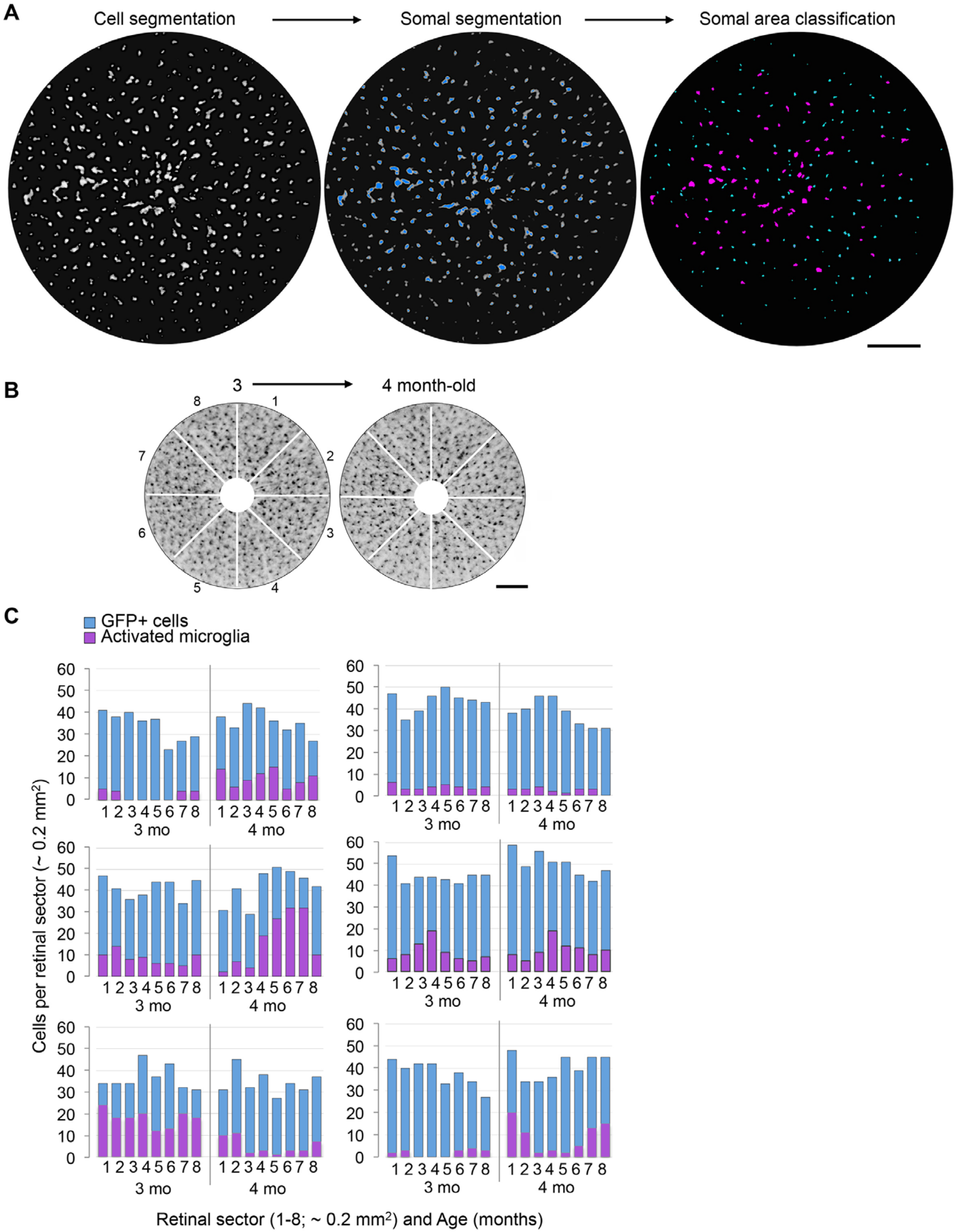

Supplementary Figure 2.

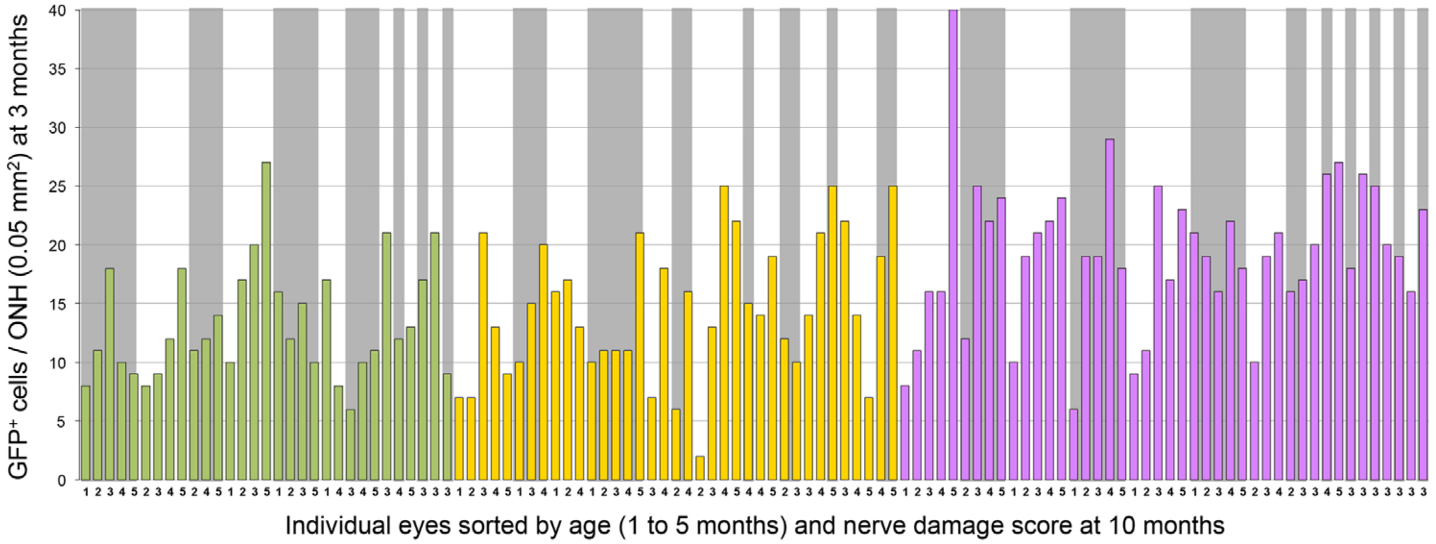

Supplementary Figure 3.
